# Multi-analytical test based on serum miRNAs and proteins quantification for ovarian cancer early detection

**DOI:** 10.1371/journal.pone.0255804

**Published:** 2021-08-05

**Authors:** Priscila D. R. Cirillo, Katia Margiotti, Marco Fabiani, Mateus C. Barros-Filho, David Sparacino, Antonella Cima, Salvatore A. Longo, Marina Cupellaro, Alvaro Mesoraca, Claudio Giorlandino

**Affiliations:** 1 Altamedica Center, Human Genetics Laboratories, Altamedica Main Center, Rome, Italy; 2 Department of Head and Neck Surgery, Hospital das Clínicas da Faculdade de Medicina da Universidade de São Paulo, São Paulo, SP, Brazil; 3 Altamedica, Department of Biochemistry, Altamedica Main Centre, Rome, Italy; 4 Altamedica, Department of Prenatal Diagnosis, Fetal-Maternal Medical Center, Rome, Italy; Gustave Roussy, FRANCE

## Abstract

Advanced ovarian cancer is one of the most lethal gynecological tumor, mainly due to late diagnoses and acquired drug resistance. MicroRNAs (miRNAs) are small-non coding RNA acting as tumor suppressor/oncogenes differentially expressed in normal and epithelial ovarian cancer and has been recognized as a new class of tumor early detection biomarkers as they are released in blood fluids since tumor initiation process. Here, we evaluated by droplet digital PCR (ddPCR) circulating miRNAs in serum samples from healthy (N = 105) and untreated ovarian cancer patients (stages I to IV) (N = 72), grouped into a discovery/training and clinical validation set with the goal to identify the best classifier allowing the discrimination between earlier ovarian tumors from health controls women. The selection of 45 candidate miRNAs to be evaluated in the discovery set was based on miRNAs represented in ovarian cancer explorative commercial panels. We found six miRNAs showing increased levels in the blood of early or late-stage ovarian cancer groups compared to healthy controls. The serum levels of miR-320b and miR-141-3p were considered independent markers of malignancy in a multivariate logistic regression analysis. These markers were used to train diagnostic classifiers comprising miRNAs (miR-320b and miR-141-3p) and miRNAs combined with well-established ovarian cancer protein markers (miR-320b, miR-141-3p, CA-125 and HE4). The miRNA-based classifier was able to accurately discriminate early-stage ovarian cancer patients from health-controls in an independent sample set (Sensitivity = 80.0%, Specificity = 70.3%, AUC = 0.789). In addition, the integration of the serum proteins in the model markedly improved the performance (Sensitivity = 88.9%, Specificity = 100%, AUC = 1.000). A cross-study validation was carried out using four data series obtained from Gene Expression Omnibus (GEO), corroborating the performance of the miRNA-based classifier (AUCs ranging from 0.637 to 0.979). The clinical utility of the miRNA model should be validated in a prospective cohort in order to investigate their feasibility as an ovarian cancer early detection tool.

## Introduction

Although ovarian cancer (OC) accounts to 2.5% of all women malignances [1] this tumor is the leading cause of gynecologic cancer mortality [2]. Because of its heterogeneous nature, ovaria n cancer early detection (stages I-II) and primary prevention and intervention has been a clinical challenge [3]. Epithelial ovarian cancer is the most common histopathological subtype, and almost 70% of patients are diagnosed at an advanced stage (III-IV), and the overall 5-years survival for FIGO (International Federation of Gynecology and Obstetrics) is only 23% [4, 5]. It is evident that efforts to optimize patients’ clinical benefits should be focused on improving the early disease detection. In fact, it has been shown that over 90% patients who are diagnosed at FIGO stage I have 5-year survival [6]. Current diagnostic methods epithelial ovarian cancer early detection mainly includes ultrasound and measurement of serum biomarkers such us carcinoembryonic antigen (CEA), cancer antigen-125 (CA-125), carbohydrate antigen 19–9 (CA19-9), and human epididymis protein 4 (HE4). Among these, CA-125 is the most common biomarker used in clinical routine of ovarian cancer management. It has been reported that CA-125 is not effective for early-stages ovarian cancer detection as it is not sufficiently specific to be used as a general population screening method, because a number of common benign conditions can cause elevation of CA-125 levels, including endometriosis, adenomyosis, ovarian cysts, uterine fibroids, renal dysfunction and hepatic disease are really [7]. Thus, there is an urgent need to develop new strategies tools able to detect ovarian cancer at earlier stages. Liquid biopsy is a minimally invasive blood-based approach that has the potential to provide relevant tumor landscape on prognosis, response to therapeutic regimens and early diagnosis [8]. The detection and characterization of circulating tumor cells (CTCs), circulating tumor DNA (ctDNA), microRNAs (miRNAs), and extracellular vesicles profiles in the human body fluids represents a promising clinical utility of liquid biopsy for cancer patients management [9–11]. MicroRNAs are a subclass of small non-coding RNA molecules (17–22 nucleotides) that negatively regulate gene expression by binding specifically to 3’ untranslated-region of their target mRNAs. One miRNA can potentially bind to hundreds of target genes and be involved in the regulation of various cellular processes, such as development, differentiation and cell proliferation [12]. They display distinct expression profiles in tumors and are able to differentiate between cancer and normal tissue, as they are released by solid tumors in human body fluids [13–15]. The purpose of this study was to identify and validate a group of circulating miRNAs in human serum able to discriminate patients with early (stages I-II) and advanced (stages III-IV) ovarian cancer from healthy patients. We have developed a high specific and sensitivity diagnostic classifier model with miR-320b, miR-141-3p, CA-125 and HE4 markers allowing the discrimination between ovarian cancer and health controls.

## Materials and methods

### Study design

**Fig 1:**
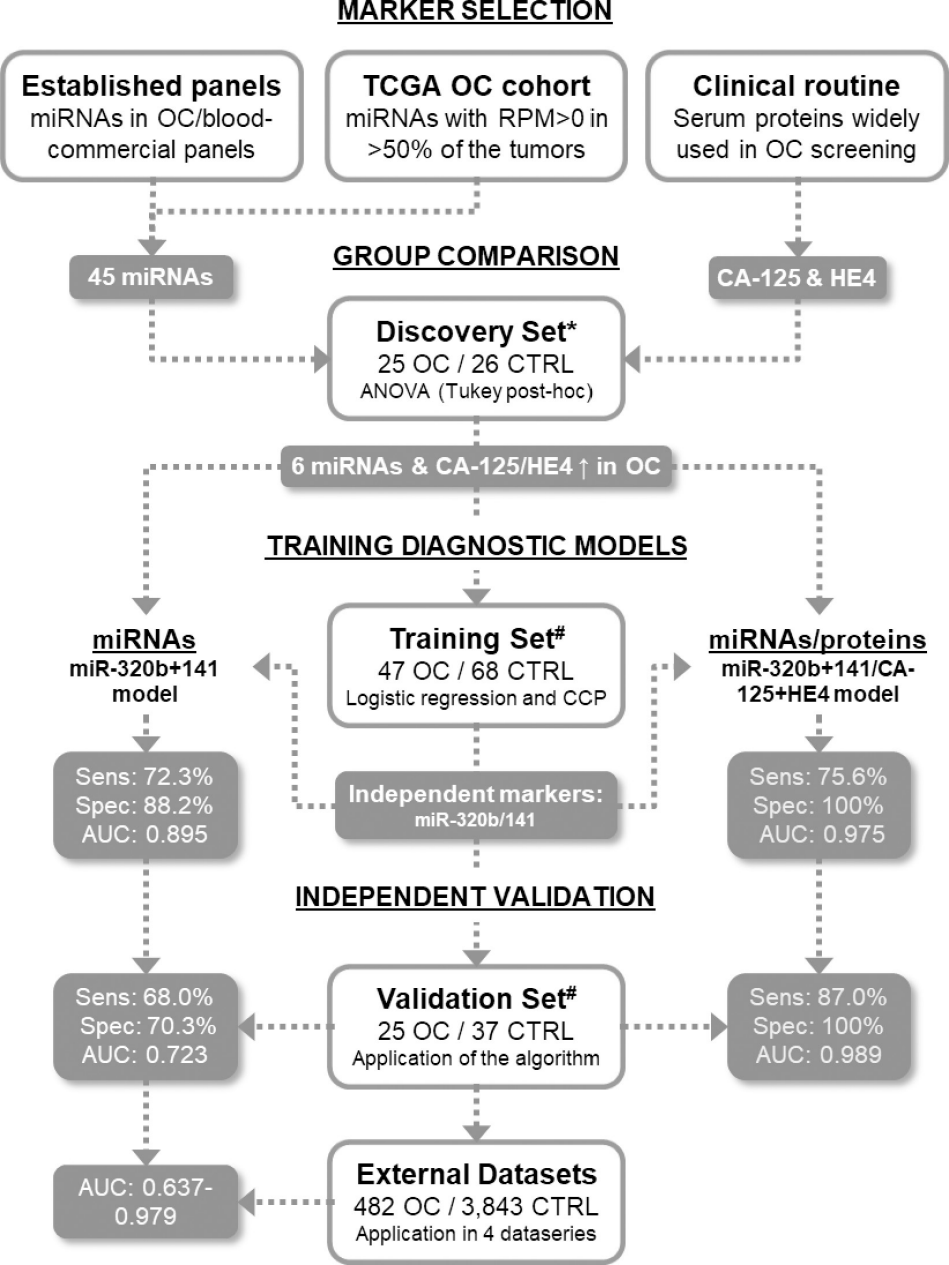
Flowchart showing the study design and main results. The potential miRNAs (n = 45) and proteins (CA-125 and HE4) serum-markers were selected and their levels were assessed and compared in the serum of OC patients and healthy controls. Both tested proteins and six miRNAs (miR-10b-5p, miR-21-5p, miR-29c-3p, miR-141-3p, miR-222-3p, and miR-320b) were overrepresented in the serum of cancer patients compared to the controls in the Discovery Set. These markers were further tested increasing the sample size in a Training Set, where miR-141-3p, miR-320b, CA-125 and HE4 were considered non-redundant independent markers in a multivariate analysis (logistic regression). Two diagnostic classifiers were designed (CCP method), using only miRNAs and combining miRNAs with proteins. The diagnostic classifiers were applied in an independent group of samples (Validation Set), confirming its diagnostic potential, especially when the miRNAs were associated with proteins. The miRNA-based model was additionally applied in four publicly available data series (External Datasets), demonstrating the diagnostic power of the model. *performed as customized miRCURY LNA plates; #performed as individual assays; OC: ovarian cancer; CTRL: healthy controls; RPM: reads per million; TCGA: The Cancer Genome Atlas; Sens: sensitivity; Spec: Specificity; AUC: area under the ROC curve; CCP: Compound Covariate Predictor.

The local Ethics Committee (Artemisia S.p.A) has approved the protocol study. All healthy donors provided written informed consent for the use of serum samples for research purposes. All serum samples from ovarian cancer patients were obtained through MTA (Material Transfer Agreement) signed consent form from the following centers: Discovery Life Sciences (Huntsville, USA), BioIVT (London, UK), Victorian Cancer Bank (Melbourne, Australia), and Wales Cancer Bank (Cardiff, Wales). Inclusion criteria for patients were: i) serous epithelial ovarian adenocarcinoma diagnosis; ii) no previous radiotherapy, chemotherapy or endocrine therapy; iii) availability of clinical data (i.e. tumor staging and subtype classification). Exclusion patients’ criteria were: i) incomplete medical history data; ii) other important organ dysfunction and iii) known synchronous neoplasia. The study design and main results found are illustrated in Fig 1.

### Ovarian cancer patients and health controls

In total, 72 OC patients and 105 Healthy Controls (HC) were enrolled in the study. The cases were stratified in a Training Set, comprising 68 healthy controls and 47 ovarian serous adenocarcinoma and a Validation Set comprising 37 healthy controls and 25 ovarian serous adenocarcinomas (Table 1). The Training Set consisted of samples evaluated by both customized plates and individual ddPCR assays (Discovery Set; 25 OCs and 26 controls) and samples evaluated exclusively by individual ddPCR assays (22 OCs and 42 controls). The Validation Set was used to confirm the results obtained in an independent fashion (tested by individual ddPCR assays).

**Table 1:** Characteristics of the patients and healthy controls in discovery/training and validation studies.

	Total (N = 177)			Discovery/Training set (N = 115)			Validation set (N = 62)		
	N	Mean	SD	N	Mean	SD	N	Mean	SD
**Healthy Controls**	105			68			37		
Age (years)		48.6	19.3		49.0	19.5		47.8	19.3
**Ovarian Cancer Patients**	72			47			25		
Age (years)		62.8	13.7		63.4	13.6		61.7	14.2
Stages I-II	30			20			10		
Stages III–IV	42			27			15		

SD = standard deviation; N = number

### Sample processing, circulating miRNA isolation and cDNA synthesis

All serum samples from ovarian serous adenocarcinoma patients which were purchased from several commercial biobanks (as cited above) were shipped frozen in 2mL tubes (dry ice) and were immediately kept at -80°C upon arrival. Serum samples from healthy-controls women were collected from blood samples in Vacutest tubes (with cloat activator) recruited in Altamedica Center (Rome) between 2018 and 2020. Blood tubes were centrifuged at 3000 rpm/4°C/10 min. Serum (1 mL) were collected to a new 2 mL tube and re-centrifuged at 16000 g/4°C/10 min. The supernatant was kept at − 80°C until miRNAs isolation. For normalization and quality control purposes, we have used cel-miR-238-3p [16] exogenous small RNA references (spike-in control) which was added in the serum samples prior miRNA isolation. cel-miR238-3p was synthesized by IDT (Integrated DNA Technologies, Coralville, USA) and delivered lyophilized. Upon arrival, spike RNA were suspended at 100μM with RNAse-free TE and storage at -80°C (stock solution). Prior to the miRNA extraction, cel-miR-238-3p was serially diluted at 400amol/μL final concentration in RNAse-free water (use solution). During the miRNA extraction process (miRNeasy Serum/Plasma Advanced Kit (cat. no. 217204, QIAGEN), 3μL of RNA spike use solution were added in each 200μL of serum sample after addition of Buffer RPL. After extraction, MiRNA samples (20μL) were kept at −80°C until cDNA synthesis by reverse transcription reaction. For the cDNA synthesis, 4μL of miRNA template were Reverse-Transcribed (RT) according to the microRNA PCR profiling using miRCURY LNA™ PCR primer sets with the QX200™ Droplet Digital™ PCR System protocol (QIAGEN). We have combined 4μL of 5x miRCURY RT Reaction Buffer, 1μL of 10x miRCURY RT Enzyme Mix, 0.2 μL of synthetic RNA spike-in UniSp6 (to be used as a reverse-transcription quality control parameter) at final concentration of 400 copies/uL and 10.8μL of RNAse free H20, totalizing a 20uL RT-PCR mix. RT-PCR reactions were transferred to the thermocycler C1000 Touch (BioRad) and followed the reverse transcriptions step (60 minutes/42°C), inactivation of reaction (5 minutes/95°C) and storage (4°C/”infinite”). cDNA samples were kept at 4°C up to 4 days or immediately used for ddPCR reactions.

### Selection of reference controls for circulating miRNA biomarker analysis

The selection of liquid biopsy-based reference controls is very challenging. Although it is highly important to control the variability caused by pre-analytical factors, there are no universal standardized normalization assays and protocols in this context [17]. An exogenous spike-in miRNAs controls from Caenorhabditis elegans (cel-miR-238-3p) was included to calibrate the inputted material in the droplet digital PCR (ddPCR) runs, as previously described [18]. In order to select a frequently detected and stable sncRNA to be used as an endogenous control in combination with the exogenous control, we assessed processed miRNA sequencing data from the exRNA Atlas (https://exrna-atlas.org/exat/datasets, search performed in January 2020) [19, 20]. Among 18 available miRNA sequencing datasets, we included three serum-based series (IDs: EXR-TTUSC1gCrGDH-AN, EXR-KJENS1sPlvS2-AN and EXR-MTEWA1cHYLo6-AN) that comprised more than 50 healthy controls cases (89, 54 and 54 samples, respectively). One-hundred and fifteen sncRNAs were detected (reads per million >0) in at least 95% of the samples in all 3 different series and were considered as endogenous control candidates. These candidates were ranked according to the standard deviation (lowest to highest) for each study and the average of the ranks was obtained. As miR-423-3p was the top ranked sncRNA, it was considered the most stable sncRNA and selected as endogenous control in our assays (S1 Table). This miRNA was already described as a suitable reference assay in ovarian tissues [21] and blood samples [22, 23]. In addition, miR-423-3p is also included among endogenous control candidates recommended by well-established panels (TaqMan™ Advanced miRNA Human Endogenous Controls, Catalog number: A34643), stating that it is consistently considered as a miRNA with relatively constant and abundant expression across tissues, cell types and treatment protocols.

### Selection of potential circulating miRNA biomarkers

To select promising miRNA as OC circulating biomarkers, we considered candidates overlapped in an ovarian cancer (solid tumor) focused commercial panel (miScript miRNA PCR Array Human Ovarian Cancer (Cat. no. 331221 MIHS-110ZA, QIAGEN) and a plasma/serum miRNA panel (miRCURY LNA miRNA Focus PCR Panel Serum/Plasma Focus miRNA, QIAGEN). The rationale of this strategy was to identify potential miRNAs involved in ovarian cancer and which could be released in serum/plasma. Despite miR-320b was only represented in the plasma/serum miRNA focused panel, we included this assay due to extensive literature findings regarding this miRNA and ovarian cancer development, resulting in 45 miRNA candidates [24, 25]. In order to avoid circulating miRNAs not expressed in the tumors itself, we checked their expression in the small RNA sequencing from ovarian cancer cohort of The Cancer Genome Atlas (TCGA) database (n = 485), obtained from University of California Santa Cruz Xena Browser (https://xenabrowser.net/ in January 2020). Forty-five miRNAs sequences were detected (reads per million> 0) in more than half of the TCGA ovarian cancer samples and were elected to customize our assay in combination with the endogenous and exogenous miRNAs (customized miRCURY LNA plate, code YCA2407, QIAGEN).

### Droplet digital PCR for miRNAs quantification

For ddPCR reaction, preliminary experiments were done to define the total cDNA and primer amount. The rational to dilute the total cDNA samples at different dilutions prior ddPCR procedure was based on target miRNA abundance to ensure clearly separation between positive and negative droplets during 1D and 2D plots ddPCR analysis [26–28] (S1 Fig): 1:10 for miR-10, 1:30 for miR-29, 1:90 for miR-21, 1:10 for miR-222-3p, 1:10 for miR-141-3p, 1:10 for miR-320, 1:10 for miR-423, 1:500 for cel-miR-238. Briefly, 21.1 μL of PCR reaction containing 0.55μL (miR-21, miR-29, miR-126) or 1.1 μL (miR-10b-5p, miR-222-3p, miR-141-3p, miR-320b, miR-423, cel-miR-238, UniSP6) of miRCURY LNA primers (QIAGEN), 9 μL of diluted cDNA and 11.1μL of 2×EvaGreen Supermix (BioRad) were loaded into the QX200 Droplet Generator cartridge (BioRad). Then, 20 μL of PCR mixture and 70 μL of Oil il for EVA Green (BioRad) were respectively loaded into the sample wells and oil wells of a disposable droplet generator cartridge (Bio-Rad). After that, droplets were generated manually by QX200 Droplet Generator device (BioRad) and 40 μL of oil+PCR reaction were carefully transferred to a 96-well PCR plate (BioRad). The cycling conditions were: 95°C for 5 min, then 40 cycles of 95°C for 30 s and 58°C for 1 min (ramping rate reduced to 1.6°C/s), and three final steps at 4°C for 5 min, 90°C for 5 min, and a 4°C indefinite hold to enhance dye stabilization. ddPCR analysis was done by using the QX Manager Standard Edition (1.2.345) software (BioRad). Detailed procedure is described elsewhere [29].

### Processing and normalization of serum miRNA levels

MicroRNAs found at very low levels in the serum of ovarian cancer patients were removed, defined by less than 1 copy/μL in more than 50% of the samples. The target miRNA absolute quantification was normalized by dividing the values of each sample by the geometric mean of the selected endogenous miRNA (miR-423-3p) and the exogenous spike-in control (cel-miR-238-3p). The normalized miRNA levels were further log_2_ converted.

### Detection of serum HE4 and CA125 protein levels

For protein detection, both the ovarian cancer patients and health-control serum were evaluated for CA-125 and HE4 levels by CMIA (chemiluminescent microparticle immunoassay) method using the automated chemiluminescence immunoassay analyzer (ARCHITECT i2000, Abbott Diagnostics, Abbott Park, IL) following clinical routine protocols.

### Group comparison

To identify miRNAs presenting differential levels in the serum of ovarian cancer patients in the Discovery set, the health-controls, Early Stages (I-II) Ovarian Cancer and Late Stages (III-IV) Ovarian cancer groups were statistically compared by ANOVA and Tukey post-hoc (*P* adjusted by multiple hypothesis using the Benjamini-Hochberg Procedure). MicroRNAs with *P* adjusted <0.05 and fold change (FC) >2 in any cancer group compared to controls were considered putative biomarkers candidates.

### Circulating miRNA/protein-based ovarian cancer diagnostic classifiers

A univariate logistic regression analysis was performed to further demonstrate the association between the miRNA levels and the risk of ovarian cancer in the Training Set. A multivariate logistic regression analysis was carried out to filter non-redundant markers. Markers presenting higher levels in the serum of cancer compared to the control group were included to design ovarian cancer diagnostic models. The classifier was applied over miRNA (ddPCR) and protein (CMIA) quantifications using the Compound Covariate Predictor method. The performance was estimated by Leave-One-Out Cross-Validation (LOOCV) (BRB array tools v.4.6.1) and using an independent set of samples (Validation Set).

### Cross-study validation

Publicly available databases were interrogated to test the circulating miRNA-model using GEO datasets (https://www.ncbi.nlm.nih.gov/gds in March 2021). The search included miRNA based-study types (Expression profiling by RT-PCR, Non-coding RNA profiling by array, Non-coding RNA profiling by genome tiling array and Non-coding RNA profiling by high throughput sequencing), with a minimum of 50 samples, using the following terms: ("Ovarian neoplasms" [Mesh]) AND (MicroRNAs [Mesh]) AND (control OR controls OR healthy OR non-cancer) AND (serum OR blood OR plasma OR circulating OR “liquid biopsy” OR biofluid). The eligible datasets included: (1) datasets with published results; (2) datasets including a minimum of 50 human samples, represented by at least 10 OCs and 10 controls (healthy or non-cancer individuals); (3) sncRNA profiling of cell-free RNA from body fluids (serum, plasma, cerebrospinal fluid and urine). The exclusion criteria were: (1) duplicate data reported in other studies; (2) lack of clinical/ histological information; (3) datasets related to letters, editorials, case reports or case series; (4) liquid biopsy studies using whole-blood RNA extraction. The available processed values (microarray and high-throughput sequencing) were used to apply the miRNA diagnostic model and the AUCs were assessed. This analysis was conducted according to the Preferred Reporting Items for Systematic Reviews and Meta-Analyses (PRISMA) [30].

### Statistical analysis

Graphpad Prism (v. 6.0; GraphPad Software Inc., La Jolla,CA, USA), IBM SPSS Statistics for Windows, Version 25.0 (IBM Corp. Armonk, NY, USA) and BRB ArrayTools (v. 4.6.1) software were employed in statistical analysis and illustrations. The null hypothesis was rejected when two-tailed *P*-value <0.05. The performance of the miRNA model and the serum proteins was estimated using sensitivity, specificity, positive predictive value (PPV) and negative predictive value (NPV).

## Results

### Identification of potential ovarian cancer serum miRNA biomarkers (Discovery Set)

**Fig 2:**
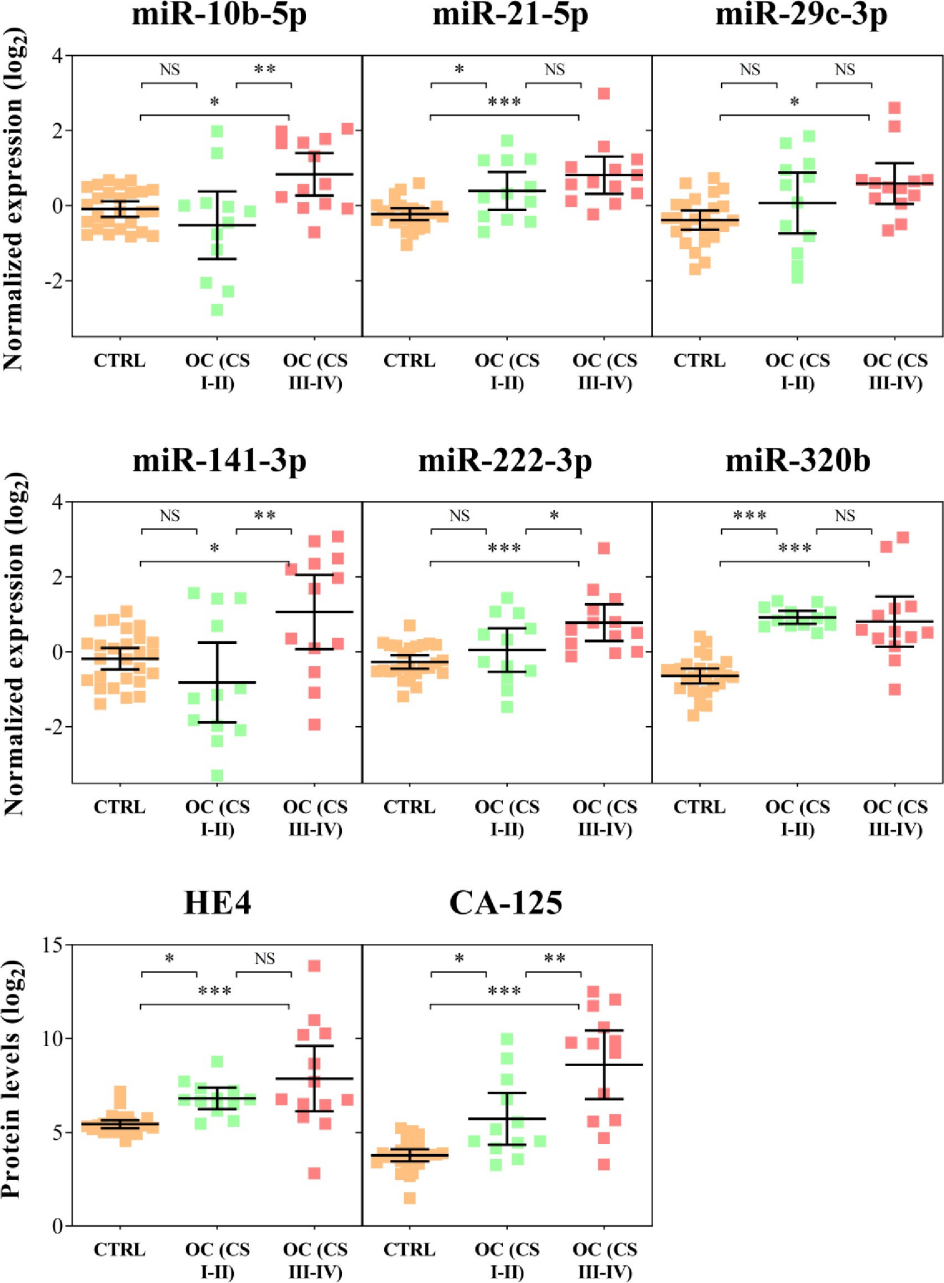
MicroRNAs and proteins that exhibited distinct levels in the serum of ovarian cancer against control cases (Discovery Set). The plot displays the mean and 95% confidence intervals of the log_2_ normalized relative quantification of the miRNAs (evaluated by miRCURY Custom ddPCR Assay) and proteins levels (measured by CMIA). For visualization purposes, the normalized miRNA levels were median-adjusted. Orange: controls (n = 26); Green: early stage (I-II) ovarian cancer (n = 12); Red: late stage (III-IV) ovarian cancer (n = 13). *P<0.05; **P<0.01; ***P<0.001 (P from Tukey post-hoc with Benjamini-Hochberg correction).

From the 45 target miRNAs assayed in the Discovery Set (51 samples analyzed by ddPCR in customized plates), 11 were frequently undetected in the serum of cancer individuals (let-7c-5p, miR-100-5p, miR-145-5p, miR-154-5p, miR-155-5p, miR-195-5p, miR-200a-3p, miR-200c-3p, miR-205-5p, miR-223-3p, and miR-375) and were subsequently removed. The biological groups were statistically compared, unveiling six miRNAs (miR-10b-5p, miR-21-5p, miR-29c-3p, miR-141-3p, miR-222-3p, miR-320b). As shown in Fig 2 and Table 2, the two miRNAs miR-21-5p and miR-320 demonstrated higher levels in serum of both early and late stages cancer groups, while miR-10b-5p, miR-29c-3p, miR-141-3p and miR-222-3p only in the late stage group (Fig 2 and Table 2). As expected, HE4 and CA-125 showed increased levels in the serum of early or late stage ovarian cancer groups compared to healthy controls (Table 2).

**Table 2:** Comparison of the ovarian cancer serum biomarkers CA-125 and HE4 and miRNAs candidates among early stage, late stage ovarian cancer patients and healthy controls groups in the Discovery Set.

Serum marker candidate	P value^#^	P value*			Fold changes		
	ES vs. LS vs. CTRL	ES vs. CTRL	LS vs. CTRL	LS vs. ES	ES vs. CTRL	LS vs. CTRL	LS vs. ES
CA-125°	**<0.0001**	**0.028**	**<0.0001**	**0.008**	**10.7**	**91.4**	**8.6**
HE4°	**<0.001**	**0.036**	**<0.001**	0.342	**2.9**	**33.8**	**11.6**
miR-10b-5p°	**0.005**	0.371	**0.029**	**0.005**	1.1	**2.1**	2.0
miR-141-3p°	**0.005**	0.364	**0.032**	**0.005**	1.0	**3.4**	**3.2**
miR-21-5p°	**<0.001**	**0.037**	**<0.001**	0.276	1.7	**2.4**	1.4
miR-222-3p°	**0.001**	0.371	**<0.001**	**0.033**	1.4	**2.4**	1.7
miR-29c-3p°	**0.019**	0.364	**0.020**	0.342	1.7	**2.2**	1.3
miR-320b°	**<0.0001**	**<0.0001**	**<0.0001**	0.909	**2.8**	**3.5**	1.2
miR-126-3p	**0.005**	0.338	**0.035**	**0.005**	1.2	1.8	1.5
miR-143-3p	**<0.001**	**0.002**	0.686	**0.002**	0.5	1.9	**4.2**
miR-125b-5p	**0.002**	**0.037**	0.160	**0.002**	0.7	**2.1**	**3.0**
miR-140-5p	**0.007**	**0.028**	0.791	**0.008**	0.7	1.2	1.7
miR-30a-5p	**0.010**	**0.037**	0.666	**0.008**	1.1	1.5	1.4
miR-133a-3p	**0.014**	**0.037**	0.686	**0.010**	0.6	**2.2**	**3.4**
miR-365a-3p	**0.016**	0.055	0.648	**0.010**	0.7	1.6	**2.2**
miR-27a-3p	**0.016**	0.089	0.488	**0.010**	0.8	1.7	**2.1**
miR-199a-5p	**0.017**	**0.034**	0.999	**0.030**	1.0	1.7	1.6
miR-221-3p	**0.040**	0.112	0.686	**0.027**	1.0	1.9	1.9
miR-199a-3p	0.051	-	-	-	1.0	1.7	1.7
miR-101-3p	0.058	-	-	-	1.7	**2.1**	1.2
miR-29a-3p	0.058	-	-	-	1.4	**2.4**	1.7
miR-26a-5p	0.058	-	-	-	1.2	**2.2**	1.9
miR-152-3p	0.058	-	-	-	1.6	**2.0**	1.2
miR-99a-5p	0.065	-	-	-	0.9	1.5	1.7
miR-30e-5p	0.074	-	-	-	1.3	1.9	1.4
let-7b-5p	0.110	-	-	-	1.4	**3.4**	**2.5**
let-7a-5p	0.144	-	-	-	1.9	1.6	0.8
miR-22-3p	0.175	-	-	-	1.9	1.8	0.9
let-7i-5p	0.242	-	-	-	1.3	1.7	1.3
miR-106b-5p	0.242	-	-	-	1.0	1.7	1.8
miR-93-5p	0.296	-	-	-	1.2	1.6	1.3
miR-16-5p	0.296	-	-	-	1.6	1.8	1.2
miR-424-5p	0.365	-	-	-	1.0	1.7	1.7
miR-15a-5p	0.381	-	-	-	1.9	1.6	0.8
miR-103a-3p	0.476	-	-	-	1.1	1.2	1.1
let-7d-5p	0.553	-	-	-	1.6	1.5	0.9

°Putative ovarian cancer serum markers; ^#^ANOVA test with Benjamini-Hochberg Procedure; *post hoc (Tukey with Benjamini-Hochberg Procedure); ES: early stage (I-II); LS: late stage (III-IV); CTRL: healthy controls. In bold, P-value <0.05.

### Developing a circulating miRNA/protein-based ovarian cancer classifier (Training set)

**Fig 3:**
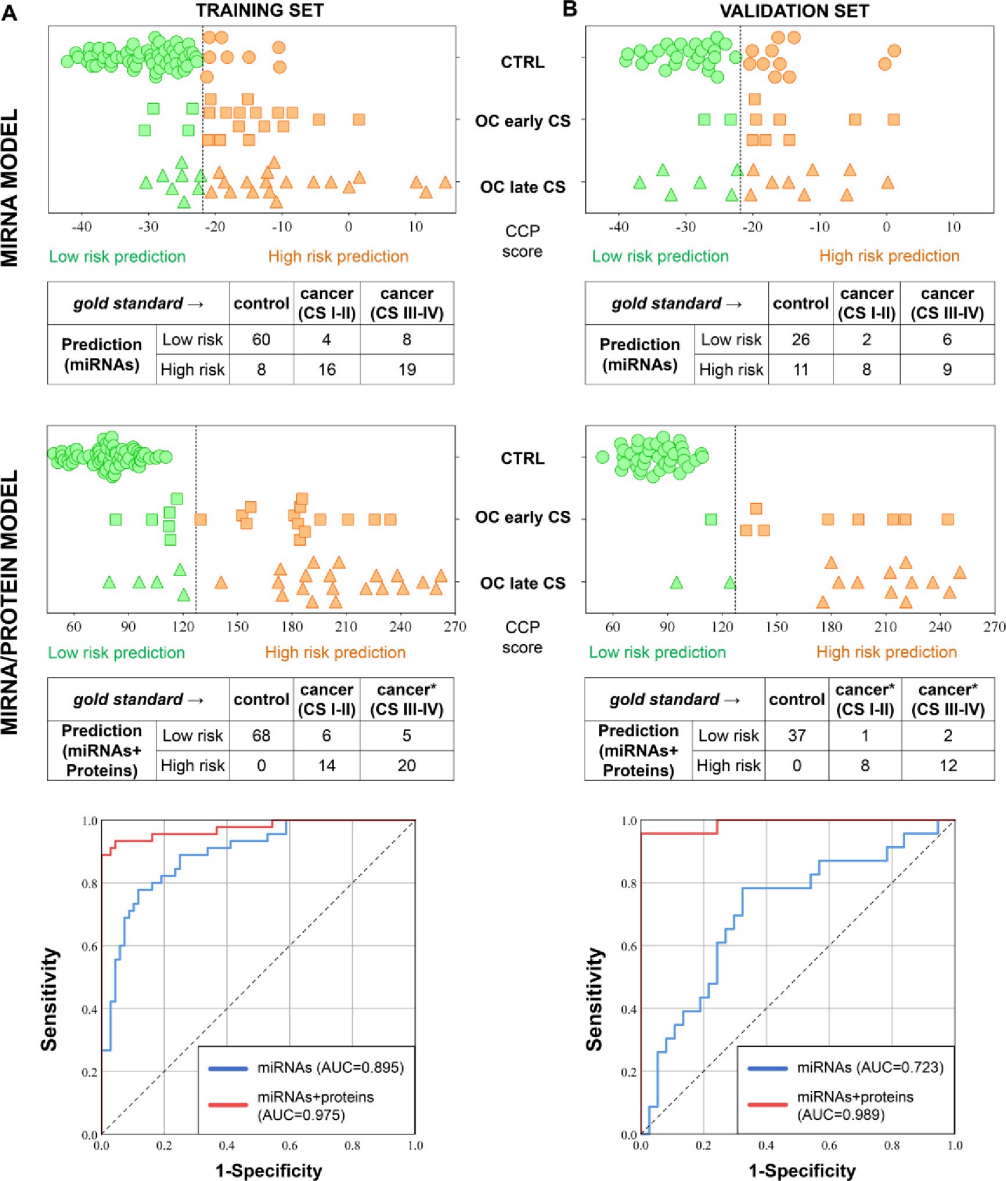
Training and validation of the ovarian cancer diagnostic models. A. Application of the miRNA-classifier (miR-141-3p and miR-320b) and the miRNA/protein-classifier (miR-320b, miR-141-3p, CA-125 and HE4) in the Training Set (n = 115) and the resulted classification and AUC. B. Application of the same classifier in the Validation Set (n = 62). The dotted line in the dot-plot represents the threshold, above of which a malignant status would be predicted (low-risk prediction in green and high-risk prediction in orange). Sample size: 105 Controls (Training n = 68; Discovery n = 37); 30 Early stages (I-II) ovarian cancer (Training n = 20; Discovery n = 10); 42 Late stages (III-IV) ovarian cancer (Training n = 27; Discovery n = 15).*HE4/CA-125 levels are not available for four OC cases (1 early and 3 late stages). CCP: Compound Covariate Predictor; AUC: area under the ROC curve; CS: clinical stages. The dotted line in the ROC curve represents the random reference (AUC = 0.5).

The six miRNAs considered as potential ovarian cancer biomarkers were additionally evaluated, using single ddPCR assays to quantify the serum miRNAs and expand the sample set from 51 to 115 (defined as Training set). All six miRNAs and the tested proteins were significantly associated with the risk of ovarian cancer in the univariate logistic regression analysis (miR-10b-5p: Odds Ratio [OR] = 1.5 [CI95% 1.0–2.4]; miR-21-5p: OR = 2.6 [CI95% 1.7–4.0]; miR-29c-3p: OR = 2.6 [CI95% 1.6–4.2]; miR-141-3p: OR = 1.7 [CI95% 1.3–2.3]; miR-222-3p: OR = 3.9 [CI95% 2.0–7.7]; miR-320b: OR = 15.1 [CI95% 5.8–39.4]; HE4: OR = 10.9 [CI95% 4.3–27.2]; CA-125: OR = 4.7 [CI95% 2.3–9.5]. A multivariate logistic regression was carried out including the six miRNAs in the model. The serum levels of miR-320b (OR = 17.7, CI95% 4.8–64.4) and miR-141-3p (OR = 2.3, CI95% 1.3–4.0) were considered independent markers of malignancy and were used to design the diagnostic classifiers. The Compound Covariate Predictor analysis was carried out using the log2-transformed protein and miRNA (relative expression value obtained after the normalization) quantifications, establishing weights to each marker and a prediction threshold. A miRNA-based classifier was trained in this dataset aiming to discriminate ovarian cancer patients from healthy controls. The application of the classifier (miRNA score = miR-320b x 8.90 + miR-141-3p x 4.14; cancer prediction threshold >-21.9), yielded a 72.3% sensitivity and 88.2% specificity in the LOOCV. By combining the miRNAs with proteins (miRNA/protein score = miR-320b x 8.90 + miR-141-3p x 4.14 + CA-125 x 12.7 + HE4 x 11.7; cancer prediction threshold >127.2), a classification with 75.6% sensitivity and 100% specificity was obtained in the LOOCV (Fig 3A and Table 3). For comparison purposes, the single use of the serum protein CA-125 (cut-off = 35U/ml) achieved 82.2% sensitivity and 88.2 specificity, while HE4 (cut-off = 100U/ml) achieved 77.8% sensitivity and 98.5 specificity in the same sample set. The miRNA/protein model presented a superior area under the ROC curve (AUC = 0.975) compared to the miRNA model and the individual use of CA-125 and HE4 (AUC = 0.895; AUC = 0.923; and 0.956, respectively).

**Table 3:** Classification performance of the miRNA and miRNA/protein-based models in distinguishing cancer from control patients in the Training Set.

Group comparison	Metric	Estimate (CI_95%_)°	
		miRNA-model	miRNA/protein-model
***OC vs*. *Healthy Control***	Sensitivity	72.3 (57.4–84.4)	75.6 (60.5–87.1)
	Specificity	88.2 (78.1–94.8)	100 (94.7–100)
	PPV	81.0 (65.9–91.4)	100 (89.7–100)
	NPV	82.2 (71.5–90.2)	86.1 (76.5–92.8)
	AUC	0.895 (0.838–0.953)	0.975 (0.945–1)
***Early-Stage OC vs*. *Healthy Control***	Sensitivity	75 (50.9–91.3)	70 (45.7–88.1)
	Specificity	88.2 (78.1–94.8)	100 (94.7–100)
	PPV	65.2 (42.7–83.6)	100 (76.8–100)
	NPV	92.3 (83–97.5)	91.9 (83.2–97)
	AUC	0.893 (0.813–0.974)	0.979 (0.943–1)
***Late-Stage OC vs*. *Healthy Control***	Sensitivity	70.4 (49.8–86.2)	80 (59.3–93.2)
	Specificity	88.2 (78.1–94.8)	100 (94.7–100)
	PPV	70.4 (49.8–86.2)	100 (83.2–100)
	NPV	88.2 (78.1–94.8)	93.2 (84.7–97.7)
	AUC	0.897 (0.83–0.964)	0.971 (0.926–1)

OC: ovarian cancer; CI_95_: 95% confidence interval (calculated by the exact Clopper-Pearson method); °Estimated by leave-one-out cross-validation; PPV = positive predictive value; NPV = negative predictive value; AUC: area under the ROC curve.

### Performance of the miRNA/protein-based classifier in an independent dataset (Validation Set)

In order to support the performance of the predictive models, the miRNAs (miR-141-3p and miR-320b) and proteins (CA-125 and HE4) were further tested in a new independent sample set (Validation Set; n = 62), adopting the same mathematical models prior described. In this Validation Set, the miRNA classifier presented a sensitivity and specificity of 68.0% and 70.3%, respectively (AUC = 0.723), while the miRNA/protein classifier presented a sensitivity and specificity of 87.0% and 100%, respectively (AUC = 0.989) (Fig 3B). Importantly, the performance of the miRNA and miRNA/protein classifiers was relatively high in the diagnostic of early-stage ovarian cancer patients (miRNA-model; sensitivity = 80.0%, specificity = 70.3%, AUC = 0.789; miRNA/protein-model; sensitivity = 88.9%, specificity = 100%, AUC = 1.000) (Table 4).

**Table 4:** Classification performance of the miRNA and miRNA/protein-based models in distinguishing cancer from control patients in the Validation set.

Group comparison	Metric	Estimate (CI_95%_)	
		miRNA-model	miRNA/protein-model
***OC vs*. *Healthy Control***	Sensitivity	68.0 (46.5–85.1)	87.0 (66.4–97.2)
	Specificity	70.3 (53–84.1)	100 (90.5–100)
	PPV	60.7 (40.6–78.5)	100 (83.2–100)
	NPV	76.5 (58.8–89.3)	92.5 (79.6–98.4)
	AUC	0.723 (0.591–0.855)	0.989 (0.967–1)
***Early Stage OC vs*. *Healthy Control***	Sensitivity	80 (44.4–97.5)	88.9 (51.8–99.7)
	Specificity	70.3 (53–84.1)	100 (90.5–100)
	PPV	42.1 (20.3–66.5)	100 (63.1–100)
	NPV	92.9 (76.5–99.1)	97.4 (86.2–99.9)
	AUC	0.789 (0.656–0.923)	1.000 (1–1)
***Late Stage OC vs*. *Healthy Control***	Sensitivity	60 (32.3–83.7)	85.7 (57.2–98.2)
	Specificity	70.3 (53–84.1)	100 (90.5–100)
	PPV	45 (23.1–68.5)	100 (73.5–100)
	NPV	81.3 (63.6–92.8)	94.9 (82.7–99.4)
	AUC	0.679 (0.506–0.852)	0.983 (0.947–1)

OC: ovarian cancer; CI_95_: 95% confidence interval (calculated by the exact Clopper-Pearson method); °Estimated by leave-one-out cross-validation; PPV = positive predictive value; NPV = negative predictive value; AUC: area under the ROC curve

### Performance of the miRNA model in independent datasets

**Fig 4:**
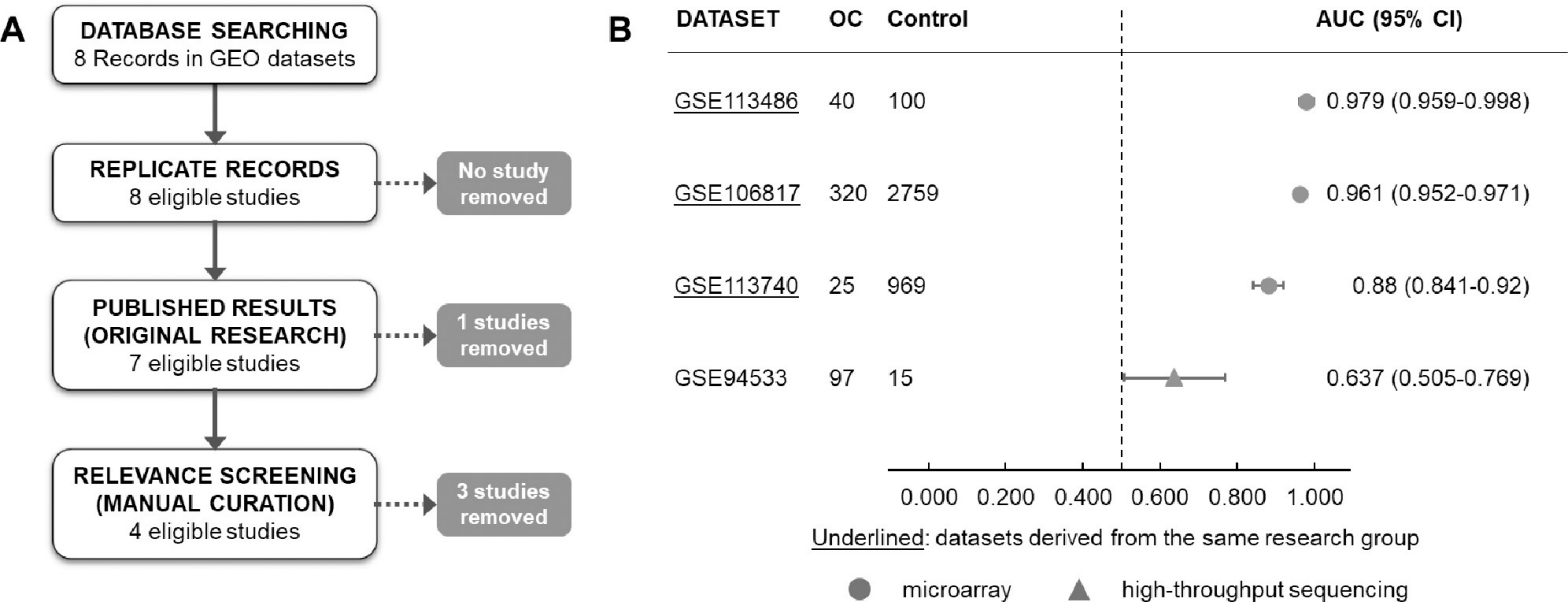
Performance of the circulating miRNA model in the publicly available datasets. A. Screening of appropriate datasets to be used in the cross-study validation step. Among eight studies found in the GEO datasets, four were eligible to test the 2-miRNA diagnostic model. B. The forest plot shows the AUCs and 95% confidence interval (CI) obtained for each study, all of them presenting the lower bound higher than 0.5.

By investigating independent available datasets from GEO, eight data series were found and four were included after employing the inclusion/exclusion criteria and curation of the published articles (S2 Table). All four series included in the cross-study validation was cell-free RNA analysis in serum, three of them based on microarray (GSE106817, GSE113486 and GSE113740) and one on high-throughput sequencing (GSE94533). The processed values (log2-transformed) were used to generate the 2-miRNA score and the ROC curve, where the AUCs varied from 0.637 to 0.979 among the datasets (Fig 4).

## Discussion

Ovarian cancer is the fifth leading cause of cancer death in women worldwide because it is usually asymptomatic in the earlier stages, and few screening tests are available. CA-125 detection was originally established to monitor patients previously diagnosed with ovarian cancer and not for tumor screening. When used as an individual marker, CA-125 is not sufficiently sensitive to detect all cases of early-stage ovarian cancer [31]. In addition, the role of CA-125 seems more relevant as a progression biomarker than for early detection in OC patients [32]. In two subsequent studies, Moore and colleagues developed an algorithm where they used a combination of CA-125, HE4 and menopausal status to predict the presence of a malignant ovarian tumor [33, 34]. Alone, HE4 had the highest sensitivity to detect ovarian cancer, especially at stage I disease. When they combined CA-125 and HE4, the predictor of malignancy was more accurate than HE4. Based on literature evidence [35], our goal was to improve the CA-125 and HE4 diagnosis power as a predictor of ovarian cancer at initial stages looking for new clinically relevant serum biomarkers. To our acknowledgement, several circulating miRNAs have been identified as potential biomarkers for cancer early detection [36–38]. However, circulating miRNAs quantification is a challenging technical approach which requires a highly sensitive method, such as, ddPCR for example [29]. In addition, a well-designed statistical analysis to establish a powerful diagnostic model was the priority of our study.

Using a strategy involving three analysis steps (Discovery, Training and Validation), we identified by ddPCR six significantly upregulated miRNAs in the serum samples of OC patients (miR-320b, miR-21-5p, miR-222-3p, miR-29c-3p, miR-10b-5p, miR-141-3p), where two of them (miR-141-3p and miR-320b) were used to design and validate a novel circulating-miRNA diagnostic model. This miRNA-based model was able to distinguish patients with ovarian cancer from healthy individuals with high accuracy (AUC = 0.895 and AUC = 0.723, in the Training and Validation sets, respectively).

Although the performance of our method in the Validation set was not high enough to corroborate the use as a single diagnostic clinical test, we showed that it could be used as a complement for the two main routinely used biomarkers (CA-125 and HE4) in the diagnosis of early stage ovarian cancer (miRNA-based model AUC = 0.789; miRNA/protein-based model AUC = 1.000).

In a recent study, Yokoi and colleagues analyzed serum miRNAs of a large cohort of 428 patients with ovarian tumors and 2759 non-cancer controls, obtaining expression profiles of 2588 miRNAs through a miRNA microarray platform [39]. The authors reported a 10-miRNA based diagnostic model (miR-320a, miR-665, miR-3184-5p, miR-6717-5p, miR-4459, miR-6076, miR-3195, miR-1275, miR-3185, and miR-4640-5p) with high accuracy in an independent cohort (99% sensitivity and 100% specificity). In our study, we considered this microarray data as one of the studies included in the cross-validation strategy to confirm the classification performance of the serum miRNA-based model (publicly available under the GSE106817 identification in GEO). Yokoi and colleagues have identified miR-320a as a relevant miRNA in their model. This miRNA belongs to “miR-320 family” and has been reported to play crucial roles in various solid and hematological tumors [40, 41]. In our study, we have identified miR-320b as an independent malignancy marker. More importantly, when combined with CA-125, HE4 and miR-141-3p our diagnosis model improved. Previous studies have shown the role of miR-320b in ovarian cancer early diagnosis and prognosis [42–44] corroborating with our findings.

In addition to miR-320b, miR-141-3p also was identified as a relevant miRNA in our diagnosis model with higher levels significantly detected among late stages (II-IV) compared to stages I-II samples (Fig 2, P<0.01). This miRNA belongs to “miR-200 family” and has been frequently detected as deregulated in a variety of OC studies [22, 45]. Interestingly, two additional miRNAs, miR-200a-3p and miR-200c-3p were undetected in our serum samples dataset, probably due to the stringent criterion adopted to avoid dealing with miRNAs found at relatively low levels in the serum. In addition, as we described above, these samples were obtained from different sample banks and we were unable to control the processing and storage steps. Although miRNAs are more stable than mRNA, various processes can influence the stability and miRNAs serum levels. Despite the importance circulating miRNAs has been gained about their role as clinical biomarkers, consistency and standardization across all diagnosis process journey are still lacking: choice of body fluids as miRNA source, pre-analytical samples processing, extraction methods, and miRNA profiling methods [46]. Besides the laboratories good practices and methods of quantification, age of the blood donors, duration of storage, differences in miRNA profile inter- and intra-populations may also account for the variability found across the studies [47, 48]. Thus, efforts are needed to establish common practices inside the laboratories to ensure circulating miRNAs can be used in clinical routine.

A limitation of our study was the healthy control subgroup. As these women were gynecological disease-free participants coming to our center for a visit routine, the mean age was around 15 years lower compared to the ovarian cancer subgroup (Table 1, 48.6 versus 62.8 mean age, respectively). In addition, the present model was trained to discriminate ovarian cancer patients from healthy control, however how the model can fit on patients with benign/borderline ovarian tumors or other tumor types is unknown. Further studies with “high-risk”populations, including older women and harboring gynecological benign conditions are needed to resolve this issue. Therefore, our healthy control dataset could explain in part the significant low levels of CA-125 and HE4 in this subgroup (Fig 2). Even with these limitations, we were able to report a new classification model using two target miRNAs as a putative diagnostic tool for ovarian cancer early detection. However, it is not expected that this signature might be self-standing, but rather that it represents a useful source of biomarkers to be added to other classifiers and predictors. Overall, our data suggest that a mathematical model with a combination of CA-125, HE4, miR-141-3p and miR-320b levels could be a suitable and accurate method for a screening of ovarian cancer, contributing to an early detection and improvement of the patient prognosis.

## Conclusion

In conclusion, we developed a multi-analytical liquid biopsy-based method using serum miRNAs that can discriminate early stages OC from healthy controls with 80.0% sensitivity and 70.3% specificity. The combination of the miRNA panel and widely used protein biomarkers could improve the OC diagnosis during early progression to 88.9% sensitivity and 100% specificity, when the patients still have a 5-year survival rate of 70–90%. Nonetheless, these data need to be validated in a large prospective study.

## Supporting information

S1 Table Small non-coding RNA selected as candidates to endogen reference controls in three data series of miRNA sequencing comprising healthy individuals. (XLSX)

S2 Table Eight datasets retrieved in GEO datasets for the cross-study validation of the miRNA-model. (XLSX)

S1 Fig Two droplet digital PCR representative experiments of cDNA and primer dilutions and 1D and 2D plots analysis on QX Manager Standard Edition (1.2.345) software (BioRad). **A.** In this experiment, a 1D plot show two health-controls samples (Sample 78 upper panel, Sample 79 below panel) used to test cDNA and primer dilution for miR-21-5p, the most abundant serum miRNA identified across ovarian cancer and health-controls subgroups. Two primer dilutions and (0.55μL and 1.1μL) and three cDNA dilutions (1:30, 1:60 and 1:90) were tested. Better separation of positive (blue) and negative (gray) droplets were obtained with primer at 0.55μL and cDNA diluted at 1:90 (red squares) for this miRNA assay. **B.** In this experiment, seven different samples (health-controls: 13, 14, 18, 19 and ovarian cancer: V8, V9, V10 samples) were used for miR-320b and cel-miR-238-3p targets. On 1D plot (left), samples can be visualized independently, and at 2D plots (right), all samples are visualized together. It is possible to observe the different miRNAs levels and separation of positive (blue) and negative (gray) droplets profiles across the samples and targets under manually defined threshold for each assay (pink lines). Red arrows show unspecific droplets excluded of the analysis using “pencil” tool. NTC: negative template control.
(TIF)
