# Supplementary figures and images for: Multi-analytical test based on serum miRNAs and proteins quantification for ovarian cancer early detection
Source: PLoS One. 2021 Aug 5;16(8):e0255804. doi: 10.1371/journal.pone.0255804 (PMC8341627; doi:10.1371/journal.pone.0255804)

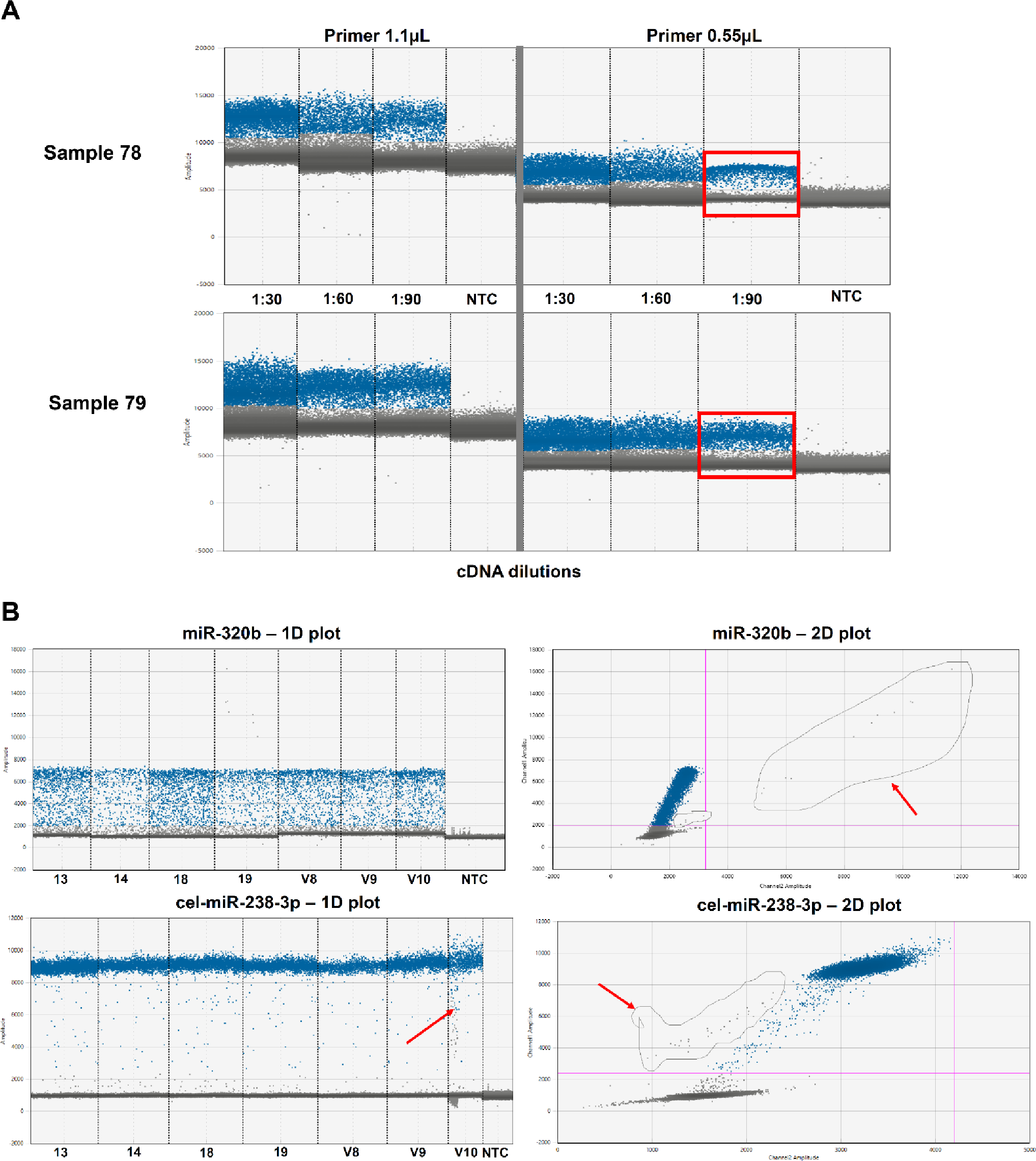

Supplement: S1 Fig — A. In this experiment, a 1D plot show two health-controls samples (Sample 78 upper panel, Sample 79 below panel) used to test cDNA and primer dilution for miR-21-5p, the most abundant serum miRNA identified across ovarian cancer and health-controls subgroups. Two primer dilutions and (0.55μL and 1.1μL) and three cDNA dilutions (1:30, 1:60 and 1:90) were tested. Better separation of positive (blue) and negative (gray) droplets were obtained with primer at 0.55μL and cDNA diluted at 1:90 (red squares) for this miRNA assay. B. In this experiment, seven different samples (health-controls: 13, 14, 18, 19 and ovarian cancer: V8, V9, V10 samples) were used for miR-320b and cel-miR-238-3p targets. On 1D plot (left), samples can be visualized independently, and at 2D plots (right), all samples are visualized together. It is possible to observe the different miRNAs levels and separation of positive (blue) and negative (gray) droplets profiles across the samples and targets under manually defined threshold for each assay (pink lines). Red arrows show unspecific droplets excluded of the analysis using “pencil” tool. NTC: negative template control. (TIF) [file pone.0255804.s003.tif]
